# H-type hypertension is a risk factor for chronic total coronary artery occlusion: a cross-sectional study from southwest China

**DOI:** 10.1186/s12872-023-03345-1

**Published:** 2023-06-16

**Authors:** Kaiyong Xiao, Zhe Xv, Yuling Xv, Jianping Wang, Lian Xiao, Zhou Kang, Jianhui Zhu, Zhongwei He, Guan Huang

**Affiliations:** 1 Department of Cardiology, Guangyuan Central Hospital, 16 Jingxiangzi, Lizhou District, Guangyuan, 628017 Sichuan China; 2 Department of Pediatric Medicine, Guangyuan Central Hospital, 16 Jingxiangzi, Lizhou District, Guangyuan, 628017 Sichuan China; 3 Sterilization Supply Center, Guangyuan Central Hospital, 16 Jingxiangzi, Lizhou District, Guangyuan, 628017 Sichuan China; 4 Department of Medical Statistics, Guangyuan Central Hospital, 16 Jingxiangzi, Lizhou District, Guangyuan, 628017 Sichuan China; 5 Medical Laboratory Center, Guangyuan Central Hospital, 16 Jingxiangzi, Lizhou District, Guangyuan, 628017 Sichuan China

**Keywords:** H-type hypertension, Chronic total coronary occlusion, Coronary occlusion, Coronary artery disease, Homocysteine, Hyperhomocysteinemia

## Abstract

**Background:**

Chronic total coronary occlusion (CTO) is serious and the "last bastion" of percutaneous coronary intervention. Hypertension and hyperhomocysteinemia (HHCY) are synergistic and significantly increase cardiovascular event risk. The relationship between H-type hypertension and CTO remains unclear; thus, this cross-sectional study investigated this potential association.

**Methods:**

Between January 2018 and June 2022, 1446 individuals from southwest China were recruited to participate in this study. CTO was defined as complete coronary artery occlusion persisting for over three months. H-type hypertension was defined as hypertension with plasma homocysteine levels ≥ 15 µmol/L. Multivariate logistic regression models were applied to assess the association between H-type hypertension and CTO. Receiver operating characteristic (ROC) curves were generated to determine the accuracy of H-type hypertension in predicting CTO.

**Results:**

Of the 1446 individuals, 397 had CTO, and 545 had H-type hypertension. After multivariate adjustment, the odds ratio (OR) for CTO in individuals with H-type hypertension was 2.3-fold higher (95% CI 1.01–5.26) than that in healthy controls. The risk of CTO is higher in individuals with H-type hypertension than in those with isolated HHCY and hypertension. The area under the ROC curve for CTO was 0.685 (95% CI, 0.653–0.717) for H-type hypertension.

**Conclusions:**

In southwest China, H-type hypertension is significantly related to the occurrence of CTO.

**Trial registration:**

This retrospective study was registered with the Chinese Clinical Trials Registry (http://www.chictr.org.cn, ChiCTR2100050519.2.2).

## Introduction

Chronic total coronary artery occlusion (CTO) is complete occlusion of a coronary artery with antegrade Thrombolysis in Myocardial Infarction (TIMI) flow grade 0 persisting for over 3 months [1]. It is one of the most complex coronary artery diseases. A registry study showed a prevalence of CTO of up to 52% [2]. Even with good collateral circulation, CTO lesions can result in persistent myocardial ischemia, producing clinical conditions such as angina pectoris, arrhythmias, and heart failure, leading to a severe adverse prognosis. Interventional treatment of CTO may be difficult secondary to regional anatomy and vessel calcification. Recanalization may require advanced approaches that then increase the cost of care while still encumbered by increased complication rates and delayed failures. Thus, CTO is thought of as the "last bastion" of percutaneous coronary intervention.

H-type hypertension is hypertension with elevated plasma homocysteine concentrations ≥ 10 µmol/L [3]; however, homocysteine levels ≥ 15 µmol/L have been reported as a cutoff value [4–6]. Some 68.3–80% of hypertensive individuals in China have H-type hypertension [7, 8]. The incidence of cardiovascular events in H-type hypertension individuals is approximately 5 times higher than in those with hypertension alone and 25 to 30 times higher than in non-hypertensive individuals [9].

In contemporary society, medical professionals have paid special attention to techniques and means of recanalizing occluded vessels, while insufficient attention has been paid to the etiology of CTO. Although traditional risk factors for CAD such as smoking, dyslipidemia, diabetes, and hypertension have received adequate attention, the study of hypertension combined with hyperhomocysteinemia (HHCY) is neglected, especially outside of China. Few studies have examined the simultaneous effects of H-type hypertension, CTO and possible multiple risk factors. However, the "Great Physician treats the untreated," and medical professionals and the public should be aware of CTO risk factors instead of determining how to deal with it only after the lesion has occurred. As no treatment reverts vasculopathy, the real value is in changing habits from an early age if a true decrease in disease occurrence or a slowing of disease progression is to occur. Therefore, early identification and management of various risk factors is particularly important. This study, which aims to investigate the potential etiology of CTO, expects to draw attention to the causes of CTO and reduce the many subsequent negative events associated with CTO.

The interactions among risk factors may be important for preventing CAD. Yet to what extent H-type hypertension affect chronic coronary artery occlusion is not known. To address this, a cross-sectional study in adults in southwest China was conducted to discern the relationship between H-type hypertension and CTO and to compare its predictive value with isolated hypertension and HHCY.

## Methods

### Study population

**Fig. 1:**
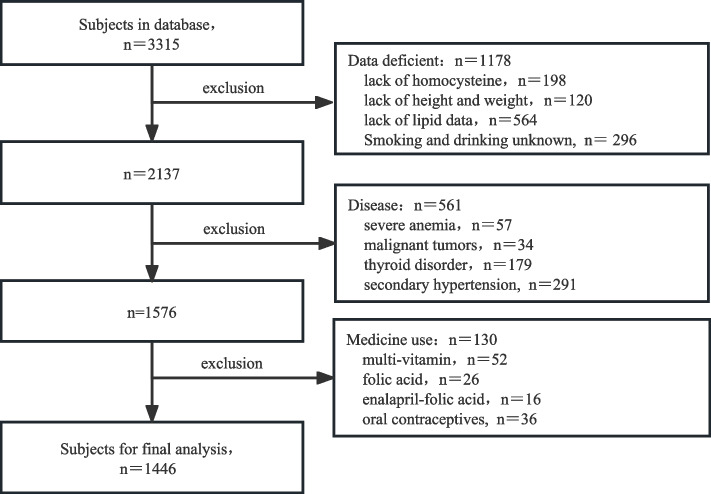
Flow diagram depicting the strategy for the selection of study participants

Medical information and data of 3315 consecutive patients who underwent coronary angiography at Guangyuan Central Hospital between January 2018 and June 2022 was reviewed. All individuals were from southwestern China. The exclusion criteria were as follows: (1) lack of primary data affecting the analysis; (2) diagnosis of abnormal thyroid function, severe anemia, malignancy, and secondary hypertension; and (3) use of folic acid or folic acid-containing preparations such as enalapril-folic acid, multivitamins [10], or oral contraceptives. Of the 3315 patients, 1869 were excluded: 198 laced homocysteine data, 120 lacked height and weight data, 564 lacked lipid data, 296 with unknown smoking and alcohol use, 57 with severe anemia, 34 found to have malignancy, 179 diagnosed with thyroid disease, 291 examined for secondary hypertension, 52 taking multivitamins, 26 taking folic acid, 16 taking enalapril-folic acid for blood pressure control, and 36 taking oral contraceptives; thus, 1446 individuals were enrolled in the study (Fig. 1). Written consent for the use of clinical data for scientific research was obtained from study individuals at admission. The study protocol was in compliance with the ethical guidelines of the Declaration of Helsinki and reviewed by the Medical Ethics Committee of Guangyuan Central Hospital (IRB protocol No. GYZXLL202118). The study was registered with the Chinese Clinical Trials Registry (http://www.chictr.org.cn, ChiCTR2100050519.2.2). There were no costs incurred by subjects that participated in the study. Dr. Zhou Kang, a professional biostatistician, participated in the data analysis.

### Data collection and measurements

All medical history and clinical information were extracted from the electronic medical record system of Guangyuan Central Hospital. Height and weight were measured at admission, and BMI was calculated as weight (kg) divided by the square of height (m). Those with a record of smoking or drinking were recorded as "yes", while those who had never been exposed to alcohol or tobacco or had been abstinent for over 6 months were recorded as "no". Peripheral venous blood samples were collected from subjects after 8–12 h of fasting. Homocysteine (Hcy), high-density lipoprotein cholesterol (HDL-C), total cholesterol (TC), apolipoprotein B (apoB), triglycerides (TG), apolipoprotein A (apoA), free fatty acids (FFA), low-density lipoprotein cholesterol (LDL-C), lipoprotein A (Lpa), glycated hemoglobin A1C (HbA1C), creatinine (Cr), cystatin C, uric acid (UA), calcium ion (Ca), troponin T (TNT), and phosphate (P) levels were measured using the same automated biochemical analyzer (ARCHITECTc16000; Abbott, Chicago, USA) at Guangyuan Central Hospital. Dyslipidemia was defined as TC ≥ 5.2 mmol/L, or TG ≥ 1.7 mmol/L, or LDL-C ≥ 3.4 mmol/L, or HDL-C < 1.0 mmol/L. Diabetes was defined as fasting glucose ≥ 7.0 mmol/L or 2-h glucose ≥ 11.1 mmol/L, HbA1C ≥ 6.5%, or current insulin or other glucose-lowering medication use. Hypertension was defined as multiple measurements of systolic blood pressure ≥ 140 mm Hg, diastolic blood pressure ≥ 90 mm Hg, or current use of anti-hypertensive medication. CKD was defined as a glomerular filtration rate (GFR) < 60 ml/min. History of ischemic stroke and cancer was obtained by self-report or results from imaging studies. Statin use was defined as oral statin therapy on admission. HHCY was defined as plasma homocysteine concentrations ≥ 15 µmol/L. H-type hypertension (H–H) was defined as hypertension with plasma homocysteine concentrations ≥ 15 µmol/L.

### Definition of CTO

All participants underwent coronary angiography (CAG) at Guangyuan Central Hospital. Two experienced cardiologists analyzed the results. CTO was defined as a complete occlusion of the coronary artery with antegrade TIMI flow grade 0 for over 3 months. Study subjects were divided into the CTO and non-CTO groups according to the CAG results. Individuals with acute total coronary artery occlusion or stenosis ≤ 99% were designated as the non-CTO group.

### Statistical analysis

Individuals were divided into four groups according to their history of hypertension and HHCY: (1) control group: without HHCY and hypertension; (2) isolated hypertension (EH) group: hypertension without HHCY; (3) isolated HHCY group: HHCY without hypertension; and (4) H-type hypertension (H–H) group: both HHCY and hypertension. The basic characteristics of the study participants are expressed as numbers and percentages for categorical variables, as the mean ± standard deviation (SD) for normally distributed continuous variables, and as the median (interquartile spacing) for variables with skewed distributions. The statistical differences among EH, HHCY, and H–H groups were tested with one-way ANOVA for continuous variables and chi-square or Fisher’s exact tests for categorical variables. Odds ratios (ORs) and 95% confidence intervals (CIs) for H-type hypertension and CTO were calculated using multivariate logistic regression models with stepwise adjustment for covariates across the four models. Model 1: unadjusted. Model 2: adjusted for sex, age, and BMI. Model 3: adjusted for sex, age, BMI, smoking status, alcohol use, diabetes, dyslipidemia, CKD, and ischemic stroke history. Model 4: Adjusted for TC, TG, apoA, apoB, HDL, LDL, LP(a), FFA, Cr, UA, cystatin C, Ca, P, HbA1C, TNT, and statin use on the basis of model 3. To determine the accuracy of the assessment of H-type hypertension in CTO, we generated receiver operating characteristic (ROC) curves. All statistical analyses were performed using R Version 4.0.3 (http://www.R-project.org, R Foundation) and Free Statistical Software version 1.7.1 (http://www.clinicalscientists.cn/freestatistics). *p* < 0.05 was considered to indicate significance.

## Results

### Characteristics of the study participants

Table 1 shows the demographic characteristics of the study participants according to CTO status. CTO was present in 397/1446 (27.5%) participants. Those with CTO tended to be older than those without (66.9 ± 10.5 VS 64.0 ± 11.5 years), were more likely to have H-type hypertension, diabetes, CKD, dyslipidemia and statin use, higher TC, apoB, LDL-C, UA, TG, LP(a), FFA, Cr, cystatin C, HbA1C, TNT levels, and lower apoA and HDL-C levels. Other baseline parameters, such as sex, BMI, smoking status, alcohol use, ischemic stroke history, and Ca and P levels, did not differ significantly (*P* > 0.05).

**Table 1:** Demographic characteristics and baseline laboratory data based on CTO

Variables	Total (*n* = 1446)	Non-CTO (*n* = 1049)	CTO (*n* = 397)	*p*
Observation object, n (%)				** < 0.001**
Control	**297 (20.5)**	**242 (23.1)**	**55 (13.9)**	
EH	**293 (20.3)**	**224 (21.4)**	**69 (17.4)**	
HHCY	**311 (21.5)**	**235 (22.4)**	**76 (19.1)**	
H–H	**545 (37.7)**	**348 (33.2)**	**197 (49.6)**	
Male, n (%)	**934 (64.6)**	**666 (63.5)**	**268 (67.5)**	**0.154**
Age, Mean ± SD	**64.8 ± 11.3**	**64.0 ± 11.5**	**66.9 ± 10.5**	** < 0.001**
BMI, Mean ± SD	**24.5 ± 3.3**	**24.5 ± 3.3**	**24.5 ± 3.2**	**0.885**
Smoking status, n (%)				**0.387**
Never smoker	**733 (50.7)**	**541 (51.6)**	**192 (48.4)**	
Current smoker	**479 (33.1)**	**346 (33)**	**133 (33.5)**	
Former smoker	**234 (16.2)**	**162 (15.4)**	**72 (18.1)**	
Alcohol use, n (%)				**0.54**
Never used alcohol	**979 (67.7)**	**704 (67.1)**	**275 (69.3)**	
Current drinker	**166 (11.5)**	**119 (11.3)**	**47 (11.8)**	
Former drinker	**301 (20.8)**	**226 (21.5)**	**75 (18.9)**	
Diabetic, n (%)	**364 (25.2)**	**235 (22.4)**	**129 (32.5)**	** < 0.001**
Dyslipidemia, n (%)	**855 (59.1)**	**582 (55.5)**	**273 (68.8)**	** < 0.001**
CKD, n (%)	**327 (25.4)**	**212 (22.9)**	**115 (31.8)**	**0.001**
Ischemic stroke history, n (%)	**97 (6.7)**	**71 (6.8)**	**26 (6.5)**	**0.882**
TC, Mean ± SD	**4.2 ± 1.0**	**4.2 ± 1.0**	**4.3 ± 1.2**	**0.004**
apoA, Mean ± SD	**1.3 ± 0.3**	**1.3 ± 0.3**	**1.2 ± 0.2**	** < 0.001**
apoB, Mean ± SD	**0.8 ± 0.3**	**0.8 ± 0.2**	**0.9 ± 0.3**	**0.004**
HDL-C, Mean ± SD	**1.2 ± 0.3**	**1.2 ± 0.3**	**1.1 ± 0.3**	** < 0.001**
LDL-C, Mean ± SD	**2.4 ± 0.8**	**2.3 ± 0.8**	**2.6 ± 0.9**	** < 0.001**
UA, Mean ± SD	**323.8 ± 104.6**	**316.4 ± 98.7**	**342.9 ± 116.7**	** < 0.001**
Ca, Mean ± SD	**2.2 ± 0.2**	**2.2 ± 0.2**	**2.2 ± 0.1**	**0.795**
P, Mean ± SD	**1.0 ± 0.3**	**1.0 ± 0.3**	**1.0 ± 0.3**	**0.793**
TG, Median (IQR)	**1.3 (1.0, 1.9)**	**1.3 (1.0, 1.8)**	**1.4 (1.0, 2.0)**	**0.021**
Lp(a), Median (IQR)	**148.0 (78.0, 305.5)**	**137.0 (72.5, 272.0)**	**176.5 (92.8, 397.0)**	** < 0.001**
FFA, Median (IQR)	**0.5 (0.3, 0.7)**	**0.5 (0.3, 0.7)**	**0.5 (0.4, 0.8)**	**0.004**
Cr, Median (IQR)	**71.0 (59.0, 84.0)**	**69.0 (58.0, 81.0)**	**75.0 (64.0, 92.0)**	** < 0.001**
Cystatin C, Median (IQR)	**1.1 (1.0, 1.4)**	**1.1 (0.9, 1.3)**	**1.2 (1.0, 1.5)**	** < 0.001**
HbA1C, Median (IQR)	**6.0 (5.6, 6.8)**	**5.9 (5.5, 6.5)**	**6.2 (5.6, 7.4)**	** < 0.001**
TNT, Median (IQR)	**23.4 (8.8, 813.8)**	**19.8 (7.4, 1070.0)**	**35.8 (12.9, 483.6)**	**0.003**
Statin use, n (%)	**175 (12.1)**	**111 (10.6)**	**64 (16.1)**	**0.004**

Abbreviations: *EH* Hypertension, *HHCY* Hyperhomocysteinemia, *H–H* H-type hypertension, *BMI* Body mass index, *TC* Total cholesterol, *ApoA* Apolipoprotein A, *ApoB* Apolipoprotein B, *HDL-C* High-density lipoprotein, *LDL-C* Low-density lipoprotein, *UA* Uric acid, *TG* Triglyceride, *Lp (a)* Lipoprotein a, *FFA* Free fatty acid, *Cr* Creatinine, *CTO* Chronic total coronary occlusion, *Ca* Serum calcium ion, *P* Phosphate, *HbA1C* Glycated hemoglobin, *CKD* Chronic kidney disease, *TNT* Troponin T

Table 2 shows the study population's demographic characteristics and baseline laboratory data based on hypertension and HHCY; 297/1446 (20.5%; mean age 60.9 ± 10.6) were healthy controls, 293/1446 (20.3%; mean age 64.4 ± 10.7) had isolated hypertension, 311/1446 (21.5%; mean age 64.0 ± 12.0) had isolated HHCY, and 545/1446 (37.7%; mean age 67.5 ± 10.9) had H-type hypertension. There were statistically significant differences between the four groups regarding sex, age, BMI, smoking status, diabetes, CKD, ischemic stroke history, dyslipidemia and statin use, and HDL-C, UA, P, TG, FFA, Cr, cystatin C, HbA1C, TNT, and apoA levels (*P* < 0.05). No significant differences were observed among alcohol use and TC, apoB, LDL-C, Ca, and Lp(a) levels (*P* > 0.05).

**Table 2:** Demographic characteristics and baseline laboratory data based on hypertension and HHCY

Variables	Total (*n* = 1446)	Control (*n* = 297)	EH (*n* = 293)	HHCY (*n* = 311)	H–H (*n* = 545)	*p*
CTO, n (%)	397 (27.5)	55 (18.5)	69 (23.5)	76 (24.4)	197 (36.1)	< 0.001
Male, n (%)	934 (64.6)	170 (57.2)	159 (54.3)	234 (75.2)	371 (68.1)	< 0.001
Age, Mean ± SD	64.8 ± 11.3	60.9 ± 10.6	64.4 ± 10.7	64.0 ± 12.0	67.5 ± 10.9	< 0.001
BMI, Mean ± SD	24.5 ± 3.3	23.9 ± 3.0	24.8 ± 3.1	23.8 ± 3.3	24.9 ± 3.4	< 0.001
Smoking status, n (%)						< 0.001
Never smoker	733 (50.7)	166 (55.9)	167 (57)	124 (39.9)	276 (50.6)	
Current smoker	479 (33.1)	94 (31.6)	78 (26.6)	138 (44.4)	169 (31)	
Former smoker	234 (16.2)	37 (12.5)	48 (16.4)	49 (15.8)	100 (18.3)	
Alcohol use, n (%)						0.071
Never drinker	979 (67.7)	204 (68.7)	207 (70.6)	194 (62.4)	374 (68.6)	
Current drinker	166 (11.5)	30 (10.1)	27 (9.2)	52 (16.7)	57 (10.5)	
Former drinker	301 (20.8)	63 (21.2)	59 (20.1)	65 (20.9)	114 (20.9)	
Diabetic, n (%)	364 (25.2)	54 (18.2)	103 (35.2)	48 (15.4)	159 (29.2)	< 0.001
Dyslipidemia, n (%)	855 (59.1)	161 (54.2)	184 (62.8)	171 (55)	339 (62.2)	0.03
CKD, n (%)	327 (25.4)	34 (12.4)	51 (19.1)	79 (29.3)	163 (34.2)	< 0.001
Ischemic stroke history, n (%)	97 (6.7)	14 (4.7)	23 (7.8)	7 (2.3)	53 (9.7)	< 0.001
TC, Mean ± SD	4.2 ± 1.0	4.2 ± 1.0	4.2 ± 1.0	4.2 ± 1.0	4.2 ± 1.1	0.990
apoA, Mean ± SD	1.3 ± 0.3	1.3 ± 0.3	1.3 ± 0.3	1.2 ± 0.2	1.2 ± 0.2	< 0.001
apoB, Mean ± SD	0.8 ± 0.3	0.8 ± 0.2	0.8 ± 0.2	0.8 ± 0.3	0.9 ± 0.3	0.184
HDL-C, Mean ± SD	1.2 ± 0.3	1.2 ± 0.3	1.2 ± 0.3	1.2 ± 0.3	1.1 ± 0.3	0.006
LDL-C, Mean ± SD	2.4 ± 0.8	2.4 ± 0.7	2.4 ± 0.8	2.4 ± 0.8	2.4 ± 0.9	0.766
UA, Mean ± SD	323.8 ± 104.6	279.1 ± 79.8	315.8 ± 90.6	333.4 ± 111.4	347.7 ± 111.5	< 0.001
Ca, Mean ± SD	2.2 ± 0.2	2.2 ± 0.2	2.2 ± 0.1	2.2 ± 0.1	2.2 ± 0.2	0.628
P, Mean ± SD	1.0 ± 0.3	1.0 ± 0.3	1.0 ± 0.3	1.0 ± 0.3	1.0 ± 0.3	0.018
TG, Median (IQR)	1.3 (1.0, 1.9)	1.2 (1.0, 1.8)	1.4 (1.1, 1.9)	1.3 (0.9, 1.7)	1.4 (1.0, 2.0)	0.002
Lp(a), Median (IQR)	148.0 (78.0, 305.5)	148.0 (73.0, 303.0)	143.0 (73.5, 292.8)	149.0 (79.0, 328.0)	149.0 (87.0, 305.0)	0.657
FFA, Median (IQR)	0.5 (0.3, 0.7)	0.5 (0.3, 0.7)	0.5 (0.4, 0.7)	0.5 (0.3, 0.7)	0.5 (0.4, 0.7)	0.024
Cr, Median (IQR)	71.0 (59.0, 84.0)	61.0 (54.0, 72.0)	68.0 (56.0, 80.0)	73.0 (61.5, 84.0)	76.0 (64.0, 94.0)	< 0.001
Cystatin C, Median (IQR)	1.1 (1.0, 1.4)	1.0 (0.9, 1.1)	1.1 (0.9, 1.3)	1.1 (1.0, 1.4)	1.3 (1.1, 1.6)	< 0.001
HbA1C, Median (IQR)	6.0 (5.6, 6.8)	5.9 (5.5, 6.4)	6.2 (5.6, 7.2)	5.8 (5.5, 6.3)	6.0 (5.6, 7.0)	< 0.001
TNT, Median (IQR)	23.4 (8.8, 813.8)	13.6 (6.4, 500.6)	14.8 (7.4, 223.4)	48.4 (9.6, 1156.0)	33.0 (11.3, 981.0)	< 0.001
Statin use, n (%)	175 (12.1)	29 (9.8)	49 (16.7)	26 (8.4)	71 (13)	0.008

Abbreviations: *EH* Hypertension, *HHCY* Hyperhomocysteinemia, *H–H* H-type hypertension, *BMI* Body mass index, *TC* Total cholesterol, *ApoA* Apolipoprotein A, *ApoB* apolipoprotein B, *HDL-C* High-density lipoprotein, *LDL-C* Low-density lipoprotein, *UA* Uric acid, *TG* Triglyceride, *Lp (a)* Lipoprotein a, *FFA* Free fatty acid, *Cr* Creatinine, *CTO* Chronic total coronary occlusion, *Ca* Serum calcium ion, *P* Phosphate, *HbA1C* Glycated hemoglobin, *CKD* Chronic kidney disease, *TNT* Troponin T

### Relationship between H-type hypertension and CTO

Table 3 shows the multiple adjusted relationships based on the hypertension, HHCY, and H–H compared with the control group. In model 1, without adjustment, the OR for CTO in H-type hypertension was 2.49 (95% CI 1.77–3.5). In model 2, the OR for CTO in H-type hypertension was 2.51 (95% CI 1.72–3.65) after adjusting for sex, age, and BMI. In model 3, the OR for CTO in H-type hypertension was 2.33 (95% CI 1.59–3.42) after adjusting for sex, age, BMI, smoking status, alcohol consumption, diabetes, dyslipidemia, CKD, and ischemic stroke history. In model 4, after adjusting for sex, age, BMI, smoking status, alcohol consumption, diabetes, dyslipidemia and statin use, CKD and ischemic stroke history, and TC, TG, apoA, apoB, HDL, LDL, LP(a), FFA, Cr, UA, cystatin C, Ca, P, HbA1C, and TNT levels, H-type hypertension remained the potent independent predictor for CTO; compared with patients without hypertension and HHCY, those with H-type hypertension were at a 2.3-fold increased risk for CTO (95% CI 1.01–5.26, *P* = 0.048).

**Table 3:** Univariate and multivariate logistic regression analysis of H-type hypertension and chronic total coronary artery occlusion

		**Model 1**		**Model 2**		**Model 3**		**Model 4**	
Variable	**event/total_%**	**crude.OR_95CI**	**crude.P_value**	**adj.OR_95CI**	**adj.P_value**	**adj.OR_95CI**	**adj.P_value**	**adj.OR_95CI**	**adj.P_value**
Control	**55/297 (18.5)**	**1(Ref)**		**1(Ref)**		**1(Ref)**		**1(Ref)**	
EH	**69/293 (23.5)**	**1.36 (0.91 ~ 2.02)**	**0.134**	**1.43 (0.93 ~ 2.18)**	**0.101**	**1.29 (0.84 ~ 1.98)**	**0.252**	**1.3 (0.55 ~ 3.06)**	**0.548**
HHCY	**76/311 (24.4)**	**1.42 (0.96 ~ 2.1)**	**0.077**	**1.48 (0.97 ~ 2.25)**	**0.07**	**1.42 (0.92 ~ 2.18)**	**0.112**	**1.65 (0.62 ~ 4.42)**	**0.318**
H–H	**197/545 (36.1)**	**2.49 (1.77 ~ 3.5)**	** < 0.001**	**2.51 (1.72 ~ 3.65)**	** < 0.001**	**2.33 (1.59 ~ 3.42)**	** < 0.001**	**2.3 (1.01 ~ 5.26)**	**0.048**
Trend.test	**397/1446 (27.5)**	**1.35 (1.22 ~ 1.5)**	** < 0.001**	**1.35 (1.2 ~ 1.51)**	** < 0.001**	**1.33 (1.18 ~ 1.5)**	** < 0.001**	**1.32 (1.03 ~ 1.71)**	**0.031**

Abbreviations: *OR* Odds ratio, *CI* Confidence interval, *EH* Hypertension, *HHCY* Hyperhomocysteinemia, *H–H* H-type hypertension

Model 1: unadjusted

Model 2: adjusted for sex, age, and BMI

Model 3: adjusted for sex, age, BMI, smoking history, alcohol use, diabetes, dyslipidemia, CKD, and ischemic stroke history

Model 4: adjusted for TC, TG, apoA, apoB, HDL, LDL, LP(a), FFA, Cr, UA, cystatin C, Ca, P, HbA1C, TNT, and statin use on the basis of model 3

**Fig. 2:**
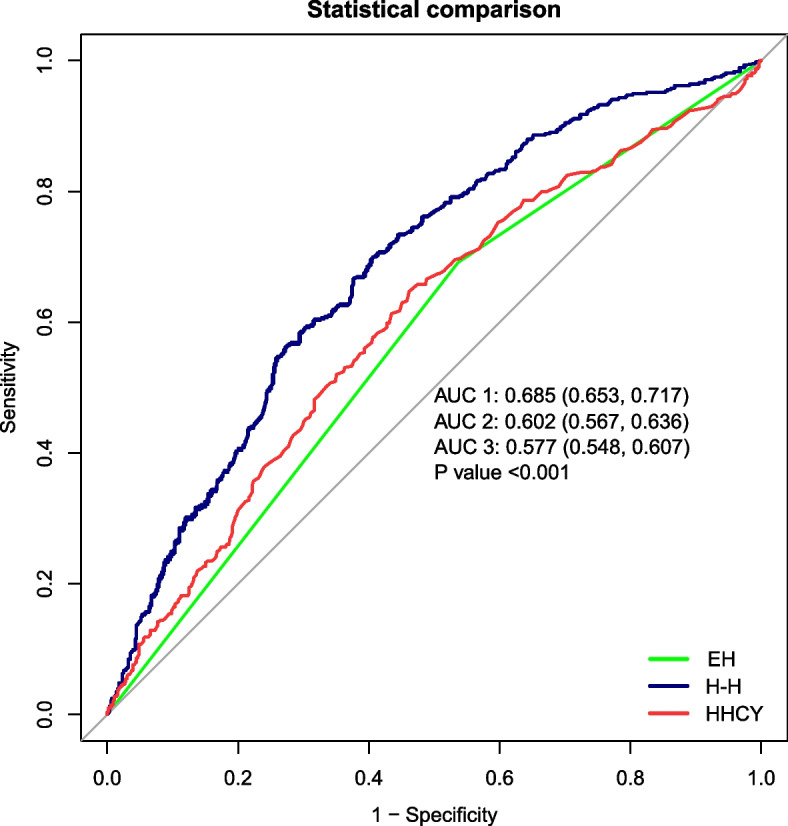
Demonstrates the diagnostic value of H-type hypertension for CTO compared to isolated HHCY and hypertension

Figure 2 demonstrates the diagnostic value of H-type hypertension for CTO compared to isolated HHCY and hypertension. The area under the ROC curve for CTO was 0.685 (95% CI, 0.653–0.717) for H-type hypertension, significantly better than 0.602 (95% CI, 0.567–0.636) for isolated HHCY and 0.577 (95% CI, 0.548–0.607) for isolated hypertension, which showed a greater predictive value (*P* < 0.001).

## Discussion

In the present cross-sectional study, H-type hypertension was associated with a higher risk than isolated hypertension or HHCY for the development of CTO in adults in southwest China. As well, the predictive value of H-type hypertension was higher than those of isolated HHCY and hypertension.

Previous studies confirmed the association of H-type hypertension with atherosclerotic diseases, such as retinal vessel abnormalities [11], carotid atherosclerotic plaques [7], carotid intima-media thickening [4], and enhanced peri-carotid adipose tissue (PCAT) [12]. When HHCY coexists with hypertension, in addition to their independent effects, their interaction tends to increase the severity of vascular disease and greatly increases the risk of ischemic stroke [13] and recurrent ischemic stroke (RIS) [14]. Some data suggested a significant interaction between stroke and plasma Hcy and hypertension [15]. However, when searching for "H-type hypertension", "hyperhomocysteinemia," and "hypertension", "coronary artery disease," and "coronary occlusion" in PubMed, Web of Science, Embase, CNKI, WANFANG MED, Baidu Scholar, Google Scholar, and the Chinese Full-text Database of Medical Journals (https://www.yiigle.com), there is scant information regarding the effect of H-type hypertension on CAD. Among individuals with H-type hypertension, coronary flow velocity reserve (CFVR), as a marker of endothelial health, found CFVR values were decreased in the H-type hypertension group [16]. In the present study, individuals with H-type hypertension were more likely to develop CTO than those with isolated HHCY and hypertension. These findings persisted even after correction for associated risk factors. These results are in line with prior data and indicate that H-type hypertension plays an important role in coronary heart disease.

CTO may be initiated through plaque disruption, followed by thrombus formation, and eventual complete occlusion of the vessel [17]. Histopathological studies showed that unstable atherosclerotic plaques manifest intraplaque angiogenesis, hemorrhage, and inflammation. Among these pathologic changes, neoangiogenesis is believed to be a main contributor to plaque vulnerability [18]. Homocysteine can exacerbate oxidative stress in hypertension, making it easier for atherosclerotic plaques to develop into vulnerable plaques and form unstable thin fibrous caps, which in combination with arterial wall ischemia, endothelial cell apoptosis, inflammatory stimulation, and intraplaque hemorrhage, eventually lead to unstable plaque rupture [19–22]. Some data link H-type hypertension and the development of atherosclerotic plaques. Additionally, arterial wall ischemia, endothelial cell apoptosis, inflammatory, and intraplaque hemorrhage can promote plaque rupture [19–22]. Compounding this, elevated Hcy and hypertension promote coagulation abnormalities and thrombosis [23].

On meta-analysis, hypertension was associated with and interacted with HCY, perhaps via targeting the vascular endothelium [7, 24–26]. Hcy promotes vasculopathy in multiple ways. For example, Hcy prevented cholesterol efflux, stimulates cytokine production, and increased aggregation of macrophages [27, 28]. Hcy stimulated oxidative stress in vascular endothelial cells, reduced nitric oxide release, caused vascular smooth muscle cell migration, and accelerated plaque formation [29]. Hcy also activated the NLRP3 inflammasome [30, 31]. Hcy inhibited vasodilation by reducing endogenous hydrogen sulfide production [32] and activated angiotensin-converting enzyme [33], with subsequent metabolic dysregulation [16, 34]. Abnormal collagen synthesis [35] and deterioration of arterial wall elastin [36–38] also play a role in this.

In animals, hypertension combined with HHCY caused arterial injury by inhibiting the Nrf2/HO-1 pathway and Nrf2 nuclear transport to increase redox stress [39], inducing inflammation via interleukin-6 (IL-6), nuclear factor-κ-gene-binding (NF-κB) p65/rela and protein kinase B (Akt1) [40–42], and by increasing perivascular collagen and coronary artery wall thickening [43].

This study has several shortcomings. First, the study was conducted at a single center in southwestern China, which may limit the general applicability of the results. Second, the cross-sectional study could only show an association between H-type hypertension and CTO and could not determine predictive power. Again, this study only included subjects who received CAG; thus, selection bias may exist. Fourth, as this was a retrospective observational study, medication history may be inaccurate. Possible contributory factors such as polymorphisms in methyl tetrahydrofolate reductase gene and blood folate and vitamin B12 were not measured. Although adjustment for confounders in multivariate logistic regression models was done, this may not have eliminated their effects. Finally, subject diagnosis at admission and anatomic details on CTO lesions were note obtained.

## Conclusions

This study revealed a correlation between H-type hypertension and CTO among a cohort of individuals treated at a medical center in southwest China. To the best of knowledge this finding is the first of its kind. It is interesting to speculate if strict homocysteine and blood pressure control would impact these risk factors. Still, the data support further research in this direction and closer attention to individuals displaying both risk factors.

## Data Availability

All data can be obtained from the corresponding author upon reasonable request.

## Abbreviations

EH: Hypertension
HHCY: Hyperhomocysteinemia
H–H: H-type hypertension
BMI: Body mass index
TC: Total cholesterol
ApoA: Apolipoprotein A
ApoB: Apolipoprotein B
HDL-C: High-density lipoprotein
LDL-C: Low-density lipoprotein
UA: Uric acid
TG: Triglyceride
Lp (a): Lipoprotein a
FFA: Free fatty acid
Cr: Creatinine
CTO: Chronic total coronary occlusion
Ca: Serum calcium ion
P: Phosphate
HbA1C: Glycated hemoglobin A1C
CKD: Chronic kidney disease
TNT: Troponin T
